# 4-Carbamoylpiperidinium phenyl­acetate hemihydrate

**DOI:** 10.1107/S1600536810047872

**Published:** 2010-11-20

**Authors:** Graham Smith, Urs D. Wermuth

**Affiliations:** a Faculty of Science and Technology, Queensland University of Technology, GPO Box 2434, Brisbane, Queensland 4001, Australia

## Abstract

The asymmetric unit of the title compound, C_6_H_13_N_2_O^+^·C_8_H_7_O_2_^−^·0.5H_2_O, comprises two isonipecotamide cations, two phenyl­acetate anions and a water mol­ecule of solvation. The hydrogen-bonding environments for both sets of ion pairs are essentially identical with the piperidinium and amide ‘ends’ of each cation involved in lateral heteromolecular hydrogen-bonded cyclic N—H⋯O associations [graph set *R*_2_^2^(11)] which incorporate a single carboxyl O-atom acceptor. These cyclic motifs enclose larger *R*_5_^5^(21) cyclic systems, forming sheet substructures which lie parallel to (101) and are linked across *b* by the single water mol­ecule *via* water O—H⋯O_c_ (c = carboxylate) associations, giving a duplex-sheet structure.

## Related literature

For structural data on isonipecotamide salts, see: Smith *et al.* (2010 ▶); Smith & Wermuth (2010*a* ▶,*b* ▶,*c* ▶). For graph-set analysis, see Etter *et al.* (1990 ▶).
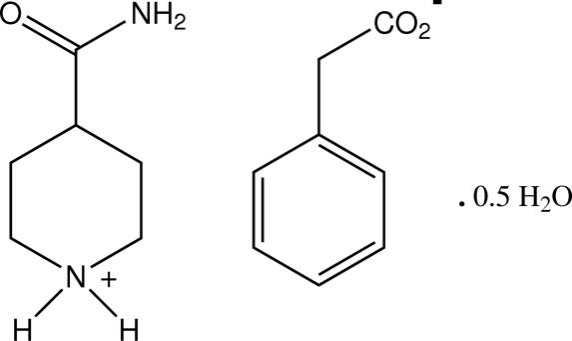


## Experimental

### 

#### Crystal data

C_6_H_13_N_2_O^+^·C_8_H_7_O_2_^−^·0.5H_2_O
*M* *_r_* = 273.33
Monoclinic, 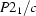

*a* = 12.3107 (9) Å
*b* = 25.214 (2) Å
*c* = 9.5402 (10) Å
β = 90.469 (9)°
*V* = 2961.2 (4) Å^3^
*Z* = 8
Mo *K*α radiation
μ = 0.09 mm^−1^
*T* = 200 K
0.50 × 0.22 × 0.20 mm

#### Data collection

Oxford Diffraction Gemini-S CCD-detector diffractometer
Absorption correction: multi-scan (*CrysAlis PRO*; Oxford Diffraction, 2009 ▶) *T*_min_ = 0.959, *T*_max_ = 0.979
21326 measured reflections
5802 independent reflections
4258 reflections with *I* > 2σ(*I*)
*R*_int_ = 0.041

#### Refinement

*R*[*F* ^2^ > 2σ(*F* ^2^)] = 0.045
*wR*(*F* ^2^) = 0.105
*S* = 1.05
5802 reflections
392 parameters
H atoms treated by a mixture of independent and constrained refinement
Δρ_max_ = 0.17 e Å^−3^
Δρ_min_ = −0.20 e Å^−3^

### 

Data collection: *CrysAlis PRO* (Oxford Diffraction, 2009 ▶); cell refinement: *CrysAlis PRO*; data reduction: *CrysAlis PRO*; program(s) used to solve structure: *SIR92* (Altomare *et al.*, 1994 ▶); program(s) used to refine structure: *SHELXL97* (Sheldrick, 2008 ▶) within *WinGX* (Farrugia, 1999 ▶); molecular graphics: *PLATON* (Spek, 2009 ▶); software used to prepare material for publication: *PLATON*.

## Supplementary Material

Crystal structure: contains datablocks global, I. DOI: 10.1107/S1600536810047872/su2230sup1.cif

Structure factors: contains datablocks I. DOI: 10.1107/S1600536810047872/su2230Isup2.hkl

Additional supplementary materials:  crystallographic information; 3D view; checkCIF report

## Figures and Tables

**Table 1:** Hydrogen-bond geometry (Å, °)

*D*—H⋯*A*	*D*—H	H⋯*A*	*D*⋯*A*	*D*—H⋯*A*
N1*C*—H11*C*⋯O13*B*^i^	0.941 (18)	1.826 (18)	2.7638 (17)	174.8 (17)
N1*C*—H12*C*⋯O13*A*^ii^	0.93 (2)	1.85 (2)	2.7322 (18)	157.9 (18)
N1*D*—H11*D*⋯O12*A*	0.924 (17)	1.876 (17)	2.7871 (17)	168.4 (16)
N1*D*—H12*D*⋯O12*B*	0.96 (2)	1.82 (2)	2.7095 (18)	153.0 (17)
N41*C*—H41*C*⋯O12*A*^iii^	0.86 (2)	2.03 (2)	2.8789 (19)	166.3 (16)
N41*C*—H42*C*⋯O41*D*^iii^	0.938 (18)	1.918 (18)	2.8480 (18)	170.8 (14)
N41*D*—H41*D*⋯O13*B*^i^	0.87 (2)	2.08 (2)	2.9177 (19)	160.6 (16)
N41*D*—H42*D*⋯O41*C*	0.913 (19)	1.917 (19)	2.8294 (18)	176.4 (18)
O1*W*—H11*W*⋯O13*A*	0.85 (2)	1.95 (2)	2.7881 (19)	171 (2)
O1*W*—H12*W*⋯O12*B*^iv^	0.84 (3)	1.99 (3)	2.8335 (19)	179 (3)

Symmetry codes: (i) 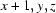; (ii) 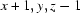; (iii) 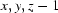; (iv) 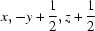.

**Fig. 1.**
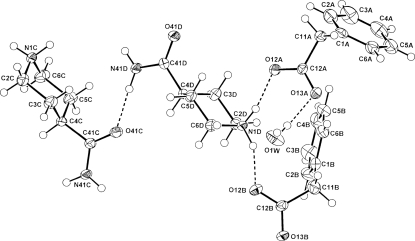
Molecular configuration and atom naming scheme for the two INIPA cations (C, D) the two phenylacetate anions (A, B) and the water molecule of solvation (O1W) in the asymmetric unit of compound (I). Inter-species hydrogen bonds are shown as dashed lines and displacement ellipsoids are drawn at the 50% probability level.

**Fig. 2.**
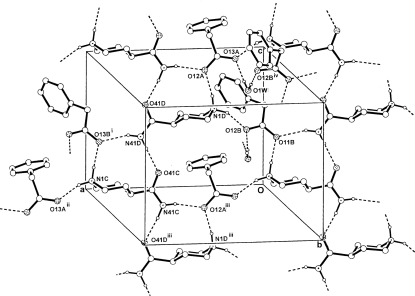
The two-dimensional hydrogen-bonded sheet substructure of compound (I) showing the R22(11) and larger R55(21) ring motifs [Non-associative H atoms have been omitted for clarity; hydrogen bonds are shown as dashed lines; for symmetry codes, see Table 1].

**Fig. 3.**
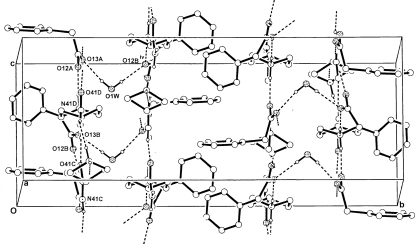
A view along the a-axis of the unit cell of compound (I), showing the water-linked duplex-sheet structure.

## supplementary crystallographic information

### Comment

The amide piperidine-4-carboxamide (isonipecotamide, INIPA) has proved to be a particularly useful synthon for the construction of hydrogen-bonded crystalline salts with a range of aromatic carboxylic acids, enabling their structure determination (Smith & Wermuth, 2010*a*, 2010*b*, 2010*c*; Smith *et al.*, 2010). The title compound from the 1:1 stoichiometric reaction of phenylacetic acid with INIPA, the hemihydrate C_6_H_13_N_2_O^+^ C_8_H_7_O_2_^-^. 0.5H_2_O, (I) was obtained and the structure is reported on herein.

In (I) the asymmetric unit contains two phenylacetate anions (*A* and *B*), two INIPA cations (*C* and *D*) and a water molecule of solvation (O1*W*) (Fig. 1). The hydrogen-bonding environments for both sets of ion pairs are essentially identical with the piperidinium and amide 'ends' of each cation involved in lateral heteromolecular cyclic hydrogen-bonded associations [graph set *R*_2_^2^(11) (Etter *et al.*, 1990)] (Table 1) which incorporate a single carboxyl O-atom acceptor (Fig. 2). The rings involve (*a*): cation *C* pyrimidinium and cation *D* amide N—H donors and cation *C* amide and anion *B* carboxyl O-atom acceptors and (*b*): cation *D* pyrimidinium and cation *C* amide N—H donors and cation *D* amide and anion *A* carboxyl O-atom acceptors. These ring motifs enclose larger cyclic systems [graph set *R*_5_^5^(21)] forming sheet substructures which lie parallel to (101) and are linked across *b* by the single water molecule *via* water *O*—*H*···O_carboxyl_ associations to give the two-dimensional duplex-sheet structure (Fig. 3).

In the two independent phenylacetate anions, the conformation of the acetate side chains are significantly different [comparative torsion angles (maximum) for C2/C6–C1–C11–C12, 95.07 (17)° (*A*) and 124.84 (17)° (*B*); C1–C11–C12–O12/O13, 90.43 (16)° (*A*) and 127.76 (16)° (*B*)].

### Experimental

The title compound was synthesized by heating together under reflux for 10 mins, 1 mmol quantities of piperidine-4-carboxamide (isonipecotamide) and phenylacetic acid in 50 ml of 50% methanol–water. After concentration to *ca* 30 ml, partial room temperature evaporation of the hot-filtered solution gave colourless plates of (I) from which a specimen was cleaved for the X-ray diffraction analysis.

### Refinement

Hydrogen atoms involved in hydrogen-bonding interactions were located in difference Fourier maps and were freely refined. Other H-atoms were included in calculated positions using a riding-model approximation [C–H = 0.93–0.98 Å] and with *U*_iso_(H) = 1.2*U*_eq_(C)].

### Crystal data

**Table:**

C_6_H_13_N_2_O^+^·C_8_H_7_O_2_^−^·0.5H_2_O	*F*(000) = 1176
*M**_r_* = 273.33	*D*_x_ = 1.226 Mg m^−^^3^
Monoclinic, *P*2_1_/*c*	Mo *K*α radiation, λ = 0.71073 Å
Hall symbol: -P 2ybc	Cell parameters from 5553 reflections
*a* = 12.3107 (9) Å	θ = 3.2–28.9°
*b* = 25.214 (2) Å	µ = 0.09 mm^−^^1^
*c* = 9.5402 (10) Å	*T* = 200 K
β = 90.469 (9)°	Prism, colourless
*V* = 2961.2 (4) Å^3^	0.50 × 0.22 × 0.20 mm
*Z* = 8	

### Data collection

**Table:**

Oxford Diffraction Gemini-S CCD-detector diffractometer	5802 independent reflections
Radiation source: Enhance (Mo) X-ray source	4258 reflections with *I* > 2σ(*I*)
graphite	*R*_int_ = 0.041
Detector resolution: 16.066 pixels mm^-1^	θ_max_ = 26.0°, θ_min_ = 3.2°
ω scans	*h* = −15→15
Absorption correction: multi-scan (*CrysAlis PRO*; Oxford Diffraction, 2009)	*k* = −31→30
*T*_min_ = 0.959, *T*_max_ = 0.979	*l* = −11→11
21326 measured reflections	

### Refinement

**Table:**

Refinement on *F*^2^	Primary atom site location: structure-invariant direct methods
Least-squares matrix: full	Secondary atom site location: difference Fourier map
*R*[*F*^2^ > 2σ(*F*^2^)] = 0.045	Hydrogen site location: inferred from neighbouring sites
*wR*(*F*^2^) = 0.105	H atoms treated by a mixture of independent and constrained refinement
*S* = 1.05	*w* = 1/[σ^2^(*F*_o_^2^) + (0.056*P*)^2^] where *P* = (*F*_o_^2^ + 2*F*_c_^2^)/3
5802 reflections	(Δ/σ)_max_ = 0.002
392 parameters	Δρ_max_ = 0.17 e Å^−^^3^
0 restraints	Δρ_min_ = −0.20 e Å^−^^3^

### Special details

**Table:**

Geometry. Bond distances, angles *etc*. have been calculated using the rounded fractional coordinates. All su's are estimated from the variances of the (full) variance-covariance matrix. The cell e.s.d.'s are taken into account in the estimation of distances, angles and torsion angles
Refinement. Refinement of *F*^2^ against ALL reflections. The weighted *R*-factor *wR* and goodness of fit *S* are based on *F*^2^, conventional *R*-factors *R* are based on *F*, with *F* set to zero for negative *F*^2^. The threshold expression of *F*^2^ > σ(*F*^2^) is used only for calculating *R*-factors(gt) *etc*. and is not relevant to the choice of reflections for refinement. *R*-factors based on *F*^2^ are statistically about twice as large as those based on *F*, and *R*- factors based on ALL data will be even larger.

### Fractional atomic coordinates and isotropic or equivalent isotropic displacement parameters (Å^2^)

**Table:**

	*x*	*y*	*z*	*U*_iso_*/*U*_eq_	
O41C	0.64163 (9)	0.15818 (5)	0.16896 (11)	0.0435 (4)	
N1C	1.02344 (12)	0.17322 (6)	0.12820 (14)	0.0270 (4)	
N41C	0.61664 (12)	0.16239 (6)	−0.06342 (14)	0.0281 (4)	
C2C	0.96731 (14)	0.22255 (7)	0.08191 (18)	0.0350 (6)	
C3C	0.84553 (13)	0.21748 (7)	0.10209 (18)	0.0328 (5)	
C4C	0.80024 (12)	0.16875 (6)	0.02592 (15)	0.0263 (5)	
C5C	0.86040 (13)	0.11923 (6)	0.07541 (17)	0.0296 (5)	
C6C	0.98170 (13)	0.12504 (7)	0.05565 (17)	0.0350 (6)	
C41C	0.67876 (13)	0.16266 (6)	0.04932 (15)	0.0263 (5)	
O41D	0.71839 (9)	0.15906 (5)	0.66969 (11)	0.0340 (4)	
N1D	0.33442 (11)	0.16040 (5)	0.62431 (14)	0.0239 (4)	
N41D	0.74212 (12)	0.15583 (6)	0.43668 (14)	0.0275 (4)	
C2D	0.38272 (13)	0.11238 (6)	0.55692 (16)	0.0268 (5)	
C3D	0.50450 (12)	0.11097 (6)	0.57817 (16)	0.0257 (5)	
C4D	0.55825 (12)	0.16182 (6)	0.52381 (15)	0.0229 (5)	
C5D	0.50677 (12)	0.21026 (6)	0.59475 (16)	0.0265 (5)	
C6D	0.38455 (13)	0.21083 (6)	0.57385 (17)	0.0281 (5)	
C41D	0.68013 (13)	0.15910 (6)	0.54975 (15)	0.0225 (5)	
O12A	0.38376 (9)	0.15741 (4)	0.90984 (10)	0.0303 (3)	
O13A	0.21718 (9)	0.17101 (4)	0.99066 (10)	0.0269 (3)	
C1A	0.34155 (14)	0.08390 (6)	1.17288 (14)	0.0267 (5)	
C2A	0.42802 (16)	0.04963 (7)	1.14988 (17)	0.0409 (6)	
C3A	0.41423 (19)	−0.00506 (8)	1.1602 (2)	0.0515 (8)	
C4A	0.31547 (19)	−0.02627 (7)	1.19230 (18)	0.0477 (7)	
C5A	0.22827 (17)	0.00717 (7)	1.21644 (18)	0.0421 (6)	
C6A	0.24171 (14)	0.06180 (7)	1.20799 (16)	0.0330 (6)	
C11A	0.35341 (13)	0.14331 (6)	1.15417 (15)	0.0262 (5)	
C12A	0.31531 (12)	0.15890 (6)	1.00715 (15)	0.0212 (5)	
O12B	0.14841 (9)	0.16112 (4)	0.47053 (11)	0.0337 (4)	
O13B	−0.02269 (9)	0.15006 (5)	0.40455 (11)	0.0344 (4)	
C1B	0.10892 (13)	0.06973 (7)	0.65695 (16)	0.0291 (5)	
C2B	0.12468 (16)	0.02946 (7)	0.55923 (18)	0.0422 (7)	
C3B	0.19546 (18)	−0.01206 (7)	0.5849 (2)	0.0488 (7)	
C4B	0.25265 (17)	−0.01453 (7)	0.70955 (19)	0.0432 (7)	
C5B	0.23861 (15)	0.02523 (7)	0.80669 (17)	0.0387 (6)	
C6B	0.16796 (14)	0.06710 (7)	0.78084 (16)	0.0329 (6)	
C11B	0.03138 (15)	0.11508 (8)	0.62690 (17)	0.0408 (6)	
C12B	0.05390 (13)	0.14407 (6)	0.48988 (15)	0.0247 (5)	
O1W	0.16998 (12)	0.24940 (7)	0.79325 (14)	0.0469 (5)	
H4C	0.81260	0.17310	−0.07480	0.0320*	
H11C	1.0116 (15)	0.1664 (6)	0.2238 (19)	0.038 (5)*	
H12C	1.0962 (19)	0.1748 (7)	0.104 (2)	0.051 (6)*	
H21C	0.98250	0.22910	−0.01620	0.0420*	
H22C	0.99450	0.25240	0.13570	0.0420*	
H31C	0.80980	0.24910	0.06660	0.0390*	
H32C	0.83010	0.21470	0.20140	0.0390*	
H41C	0.5472 (17)	0.1585 (6)	−0.0576 (18)	0.035 (5)*	
H42C	0.6465 (16)	0.1648 (6)	−0.1533 (19)	0.040 (5)*	
H51C	0.84530	0.11320	0.17370	0.0360*	
H52C	0.83450	0.08870	0.02290	0.0360*	
H61C	1.01830	0.09390	0.09270	0.0420*	
H62C	0.99740	0.12740	−0.04360	0.0420*	
H4D	0.54510	0.16430	0.42260	0.0270*	
H11D	0.3410 (14)	0.1582 (6)	0.7207 (18)	0.031 (5)*	
H12D	0.2590 (17)	0.1610 (7)	0.5982 (19)	0.043 (5)*	
H21D	0.36610	0.11270	0.45740	0.0320*	
H22D	0.35080	0.08070	0.59700	0.0320*	
H31D	0.53420	0.08060	0.52920	0.0310*	
H32D	0.52090	0.10690	0.67720	0.0310*	
H41D	0.8121 (17)	0.1539 (7)	0.4489 (18)	0.036 (5)*	
H42D	0.7104 (16)	0.1581 (7)	0.350 (2)	0.046 (6)*	
H51D	0.52350	0.20960	0.69430	0.0320*	
H52D	0.53750	0.24240	0.55580	0.0320*	
H61D	0.35380	0.24050	0.62460	0.0340*	
H62D	0.36780	0.21560	0.47510	0.0340*	
H2A	0.49580	0.06340	1.12740	0.0490*	
H3A	0.47310	−0.02740	1.14510	0.0620*	
H4A	0.30680	−0.06280	1.19790	0.0570*	
H5A	0.16070	−0.00700	1.23830	0.0510*	
H6A	0.18310	0.08400	1.22610	0.0400*	
H11A	0.42880	0.15350	1.16740	0.0320*	
H12A	0.31030	0.16170	1.22360	0.0320*	
H2B	0.08670	0.03050	0.47460	0.0510*	
H3B	0.20450	−0.03840	0.51780	0.0590*	
H4B	0.29990	−0.04250	0.72770	0.0520*	
H5B	0.27710	0.02400	0.89100	0.0460*	
H6B	0.16020	0.09370	0.84760	0.0400*	
H11B	0.03570	0.14030	0.70350	0.0490*	
H12B	−0.04220	0.10130	0.62380	0.0490*	
H11W	0.1841 (19)	0.2232 (9)	0.846 (2)	0.065 (7)*	
H12W	0.163 (2)	0.2760 (11)	0.846 (3)	0.086 (9)*	

### Atomic displacement parameters (Å^2^)

**Table:**

	*U*^11^	*U*^22^	*U*^33^	*U*^12^	*U*^13^	*U*^23^
O41C	0.0191 (6)	0.0934 (10)	0.0181 (6)	−0.0034 (6)	0.0012 (5)	0.0025 (6)
N1C	0.0149 (7)	0.0434 (9)	0.0226 (7)	−0.0017 (6)	0.0017 (6)	0.0040 (6)
N41C	0.0144 (7)	0.0510 (9)	0.0189 (7)	0.0010 (7)	0.0001 (6)	−0.0011 (6)
C2C	0.0265 (10)	0.0390 (10)	0.0395 (10)	−0.0072 (8)	−0.0041 (8)	0.0141 (8)
C3C	0.0237 (9)	0.0306 (9)	0.0442 (10)	0.0037 (7)	−0.0016 (8)	0.0081 (8)
C4C	0.0160 (8)	0.0466 (10)	0.0163 (7)	0.0002 (7)	−0.0002 (6)	0.0043 (7)
C5C	0.0229 (9)	0.0359 (10)	0.0301 (8)	−0.0013 (7)	−0.0014 (7)	−0.0096 (7)
C6C	0.0215 (9)	0.0496 (11)	0.0338 (9)	0.0079 (8)	0.0002 (7)	−0.0102 (8)
C41C	0.0188 (8)	0.0401 (10)	0.0200 (8)	0.0012 (7)	0.0001 (6)	0.0004 (7)
O41D	0.0217 (6)	0.0623 (8)	0.0181 (5)	−0.0019 (6)	−0.0009 (5)	0.0038 (5)
N1D	0.0143 (7)	0.0379 (8)	0.0195 (7)	−0.0006 (6)	−0.0009 (5)	0.0042 (6)
N41D	0.0155 (7)	0.0484 (9)	0.0185 (7)	0.0002 (7)	0.0013 (6)	−0.0012 (6)
C2D	0.0230 (9)	0.0317 (9)	0.0258 (8)	−0.0058 (7)	0.0010 (7)	−0.0035 (7)
C3D	0.0203 (9)	0.0261 (8)	0.0307 (8)	0.0001 (7)	0.0021 (7)	−0.0016 (7)
C4D	0.0172 (8)	0.0339 (9)	0.0175 (7)	−0.0010 (7)	0.0013 (6)	0.0038 (6)
C5D	0.0221 (9)	0.0248 (8)	0.0326 (8)	−0.0023 (7)	−0.0001 (7)	0.0062 (7)
C6D	0.0220 (9)	0.0297 (9)	0.0327 (8)	0.0031 (7)	−0.0004 (7)	0.0077 (7)
C41D	0.0221 (8)	0.0268 (8)	0.0187 (8)	−0.0020 (7)	0.0018 (6)	0.0028 (6)
O12A	0.0181 (6)	0.0532 (7)	0.0197 (5)	0.0004 (5)	0.0027 (5)	0.0023 (5)
O13A	0.0175 (6)	0.0357 (6)	0.0274 (6)	0.0027 (5)	0.0030 (5)	0.0035 (5)
C1A	0.0293 (9)	0.0371 (9)	0.0138 (7)	0.0067 (8)	−0.0014 (6)	0.0020 (7)
C2A	0.0361 (11)	0.0503 (12)	0.0363 (10)	0.0116 (9)	0.0064 (8)	0.0089 (9)
C3A	0.0600 (15)	0.0461 (12)	0.0486 (12)	0.0266 (11)	0.0090 (10)	0.0079 (9)
C4A	0.0755 (16)	0.0314 (11)	0.0361 (10)	0.0096 (10)	−0.0034 (10)	0.0064 (8)
C5A	0.0494 (12)	0.0411 (11)	0.0358 (10)	−0.0062 (10)	−0.0026 (9)	0.0088 (8)
C6A	0.0324 (10)	0.0367 (10)	0.0298 (9)	0.0061 (8)	0.0001 (7)	0.0036 (7)
C11A	0.0236 (9)	0.0359 (9)	0.0192 (8)	−0.0002 (7)	−0.0005 (6)	−0.0017 (7)
C12A	0.0193 (8)	0.0223 (8)	0.0220 (8)	−0.0036 (6)	0.0007 (6)	−0.0012 (6)
O12B	0.0200 (6)	0.0432 (7)	0.0377 (6)	−0.0031 (5)	−0.0059 (5)	0.0081 (5)
O13B	0.0167 (6)	0.0597 (8)	0.0268 (6)	−0.0025 (5)	−0.0036 (5)	0.0070 (5)
C1B	0.0238 (9)	0.0388 (10)	0.0248 (8)	−0.0061 (7)	0.0030 (7)	0.0071 (7)
C2B	0.0518 (13)	0.0444 (12)	0.0303 (9)	−0.0107 (10)	−0.0115 (9)	0.0002 (8)
C3B	0.0715 (15)	0.0283 (10)	0.0465 (11)	−0.0041 (10)	−0.0065 (11)	−0.0039 (9)
C4B	0.0502 (13)	0.0329 (10)	0.0466 (11)	0.0020 (9)	0.0024 (9)	0.0140 (9)
C5B	0.0357 (11)	0.0533 (12)	0.0270 (9)	−0.0009 (9)	−0.0037 (8)	0.0107 (8)
C6B	0.0304 (10)	0.0465 (11)	0.0219 (8)	−0.0027 (8)	0.0025 (7)	−0.0002 (7)
C11B	0.0275 (10)	0.0645 (13)	0.0306 (9)	0.0098 (9)	0.0064 (8)	0.0111 (9)
C12B	0.0168 (8)	0.0322 (9)	0.0251 (8)	0.0042 (7)	0.0004 (7)	−0.0016 (7)
O1W	0.0625 (10)	0.0482 (9)	0.0300 (7)	0.0147 (8)	−0.0042 (7)	0.0048 (7)

### Geometric parameters (Å, °)

**Table:**

O41C—C41C	1.2382 (18)	C5D—C6D	1.516 (2)
O41D—C41D	1.2338 (18)	C2D—H21D	0.9700
O12A—C12A	1.2594 (18)	C2D—H22D	0.9700
O13A—C12A	1.2547 (18)	C3D—H31D	0.9700
O12B—C12B	1.2554 (19)	C3D—H32D	0.9700
O13B—C12B	1.2498 (19)	C4D—H4D	0.9800
O1W—H11W	0.85 (2)	C5D—H52D	0.9700
O1W—H12W	0.84 (3)	C5D—H51D	0.9700
N1C—C6C	1.488 (2)	C6D—H61D	0.9700
N1C—C2C	1.488 (2)	C6D—H62D	0.9700
N41C—C41C	1.315 (2)	C1A—C6A	1.393 (2)
N1C—H12C	0.93 (2)	C1A—C2A	1.390 (3)
N1C—H11C	0.941 (18)	C1A—C11A	1.516 (2)
N41C—H41C	0.86 (2)	C2A—C3A	1.393 (3)
N41C—H42C	0.938 (18)	C3A—C4A	1.365 (3)
N1D—C2D	1.496 (2)	C4A—C5A	1.386 (3)
N1D—C6D	1.495 (2)	C5A—C6A	1.390 (3)
N41D—C41D	1.329 (2)	C11A—C12A	1.527 (2)
N1D—H12D	0.96 (2)	C2A—H2A	0.9300
N1D—H11D	0.924 (17)	C3A—H3A	0.9300
N41D—H42D	0.913 (19)	C4A—H4A	0.9300
N41D—H41D	0.87 (2)	C5A—H5A	0.9300
C2C—C3C	1.519 (2)	C6A—H6A	0.9300
C3C—C4C	1.530 (2)	C11A—H11A	0.9700
C4C—C5C	1.525 (2)	C11A—H12A	0.9700
C4C—C41C	1.522 (2)	C1B—C11B	1.515 (3)
C5C—C6C	1.514 (2)	C1B—C6B	1.384 (2)
C2C—H21C	0.9700	C1B—C2B	1.393 (2)
C2C—H22C	0.9700	C2B—C3B	1.383 (3)
C3C—H31C	0.9700	C3B—C4B	1.378 (3)
C3C—H32C	0.9700	C4B—C5B	1.377 (2)
C4C—H4C	0.9800	C5B—C6B	1.389 (3)
C5C—H52C	0.9700	C11B—C12B	1.525 (2)
C5C—H51C	0.9700	C2B—H2B	0.9300
C6C—H62C	0.9700	C3B—H3B	0.9300
C6C—H61C	0.9700	C4B—H4B	0.9300
C2D—C3D	1.512 (2)	C5B—H5B	0.9300
C3D—C4D	1.535 (2)	C6B—H6B	0.9300
C4D—C5D	1.536 (2)	C11B—H12B	0.9700
C4D—C41D	1.520 (2)	C11B—H11B	0.9700
			
O1W···O13A	2.7881 (19)	C12B···H2B	2.9000
O1W···C2C^i^	3.272 (2)	C12B···H22C^i^	3.0500
O1W···O12B^ii^	2.8335 (19)	C12B···H11C^vi^	2.648 (18)
O1W···N1C^i^	3.081 (2)	C41C···H42D	2.894 (19)
O12A···N1D	2.7871 (17)	C41C···H52D^iv^	2.9600
O12A···N41C^iii^	2.8789 (19)	C41D···H31C^ii^	2.8200
O12B···N1D	2.7095 (18)	C41D···H42C^iii^	2.870 (18)
O12B···O1W^iv^	2.8335 (19)	H2A···H11A	2.4500
O12B···C6D	3.309 (2)	H2B···C5A^vii^	3.0900
O12B···C2D	3.236 (2)	H2B···C2B^xii^	3.0200
O13A···C6B	3.349 (2)	H2B···C12B	2.9000
O13A···C6C^v^	3.188 (2)	H4A···O41D^viii^	2.7500
O13A···O1W	2.7881 (19)	H4C···O41D^vii^	2.7100
O13A···N1C^v^	2.7322 (18)	H4C···H62C	2.5700
O13B···N41D^vi^	2.9177 (19)	H4C···H42C	2.1800
O13B···N1C^vi^	2.7638 (17)	H4C···H21C	2.5800
O13B···C6C^vi^	3.389 (2)	H4D···H42D	2.1600
O41C···N41D	2.8294 (18)	H4D···O41C	2.7100
O41D···N41C^iii^	2.8480 (18)	H4D···H62D	2.5900
O1W···H61D	2.8000	H4D···H21D	2.5800
O1W···H12A^iv^	2.9100	H6A···H12A	2.5100
O1W···H12C^i^	2.777 (19)	H6A···H61C^v^	2.4000
O1W···H22C^i^	2.6200	H6B···H11B	2.3600
O12A···H41C^iii^	2.03 (2)	H6B···O13A	2.4800
O12A···H11D	1.876 (17)	H6B···C12A	2.9400
O12B···H12W^iv^	1.99 (3)	H6B···C6C^v^	3.0800
O12B···H12D	1.82 (2)	H6B···H62C^v^	2.4200
O12B···H11C^vi^	2.886 (18)	H11A···H2A	2.4500
O13A···H11W	1.95 (2)	H11A···O41C^iii^	2.6200
O13A···H6B	2.4800	H11B···H62C^v^	2.4800
O13A···H12C^v^	1.85 (2)	H11B···H6B	2.3600
O13B···H41D^vi^	2.08 (2)	H11C···O13B^ix^	1.826 (18)
O13B···H11C^vi^	1.826 (18)	H11C···O12B^ix^	2.886 (18)
O13B···H51C^vi^	2.8800	H11C···H32C	2.5500
O41C···H32C	2.7400	H11C···H51C	2.4900
O41C···H51C	2.7500	H11C···C12B^ix^	2.648 (18)
O41C···H4D	2.7100	H11D···C12A	2.754 (17)
O41C···H42D	1.917 (19)	H11D···O12A	1.876 (17)
O41C···H11A^vii^	2.6200	H11W···C12A	2.75 (2)
O41D···H51D	2.7300	H11W···O13A	1.95 (2)
O41D···H4A^viii^	2.7500	H12A···H6A	2.5100
O41D···H42C^iii^	1.918 (18)	H12A···O1W^ii^	2.9100
O41D···H31C^ii^	2.7600	H12C···C12A^xi^	2.89 (2)
O41D···H4C^iii^	2.7100	H12C···O1W^x^	2.777 (19)
O41D···H32D	2.7700	H12C···O13A^xi^	1.85 (2)
N1C···O13B^ix^	2.7638 (17)	H12D···C12B	2.75 (2)
N1C···O1W^x^	3.081 (2)	H12D···O12B	1.82 (2)
N1C···O13A^xi^	2.7322 (18)	H12D···C1B	3.007 (19)
N1D···O12A	2.7871 (17)	H12D···C11B	3.05 (2)
N1D···O12B	2.7095 (18)	H12W···C12B^ii^	2.79 (3)
N41C···O12A^vii^	2.8789 (19)	H12W···O12B^ii^	1.99 (3)
N41C···O41D^vii^	2.8480 (18)	H21C···H4C	2.5800
N41D···O41C	2.8294 (18)	H21C···H62C	2.5800
N41D···O13B^ix^	2.9177 (19)	H21D···C6A^vii^	3.1000
N41C···H52D^iv^	2.8300	H21D···C1A^vii^	2.8200
N41C···H51D^vii^	2.8300	H21D···C11A^vii^	3.0000
N41D···H32C	2.9100	H21D···H4D	2.5800
N41D···H31C^ii^	2.8200	H22C···C12B^x^	3.0500
C2B···C5A^vii^	3.564 (3)	H22C···O1W^x^	2.6200
C2B···C2B^xii^	3.585 (3)	H22D···C4B	2.9000
C2C···O1W^x^	3.272 (2)	H22D···C5B	2.8100
C2D···C6B	3.599 (2)	H22D···C6B	2.8900
C2D···O12B	3.236 (2)	H22D···C3B	3.0200
C3B···C5A^vii^	3.575 (3)	H22D···C1B	3.0500
C5A···C2B^iii^	3.564 (3)	H22D···C2B	3.0900
C5A···C3B^iii^	3.575 (3)	H31C···C41D^iv^	2.8200
C6B···O13A	3.349 (2)	H31C···O41D^iv^	2.7600
C6B···C2D	3.599 (2)	H31C···N41D^iv^	2.8200
C6C···O13A^xi^	3.188 (2)	H32C···O41C	2.7400
C6C···O13B^ix^	3.389 (2)	H32C···N41D	2.9100
C6D···C12A^iv^	3.451 (2)	H32C···H11C	2.5500
C6D···O12B	3.309 (2)	H32C···H42D	2.5000
C12A···C6D^ii^	3.451 (2)	H32C···H51C	2.5800
C1A···H21D^iii^	2.8200	H32D···H51D	2.5900
C1B···H22D	3.0500	H32D···O41D	2.7700
C1B···H12D	3.007 (19)	H41C···C12A^vii^	2.93 (2)
C2B···H22D	3.0900	H41C···O12A^vii^	2.03 (2)
C2B···H2B^xii^	3.0200	H41D···O13B^ix^	2.08 (2)
C3B···H22D	3.0200	H41D···C12B^ix^	3.01 (2)
C4B···H22D	2.9000	H42C···O41D^vii^	1.918 (18)
C4B···H51C^xiii^	2.9800	H42C···H51D^vii^	2.3800
C5A···H2B^iii^	3.0900	H42C···C41D^vii^	2.870 (18)
C5B···H22D	2.8100	H42C···H4C	2.1800
C6A···H21D^iii^	3.1000	H42D···H4D	2.1600
C6A···H61C^v^	3.0600	H42D···C41C	2.894 (19)
C6B···H22D	2.8900	H42D···O41C	1.917 (19)
C6B···H62C^v^	3.1000	H42D···H32C	2.5000
C6C···H6B^xi^	3.0800	H51C···H32C	2.5800
C11A···H61D^ii^	2.9400	H51C···O13B^ix^	2.8800
C11A···H21D^iii^	3.0000	H51C···C4B^xiii^	2.9800
C11B···H12D	3.05 (2)	H51C···O41C	2.7500
C12A···H12C^v^	2.89 (2)	H51C···H11C	2.4900
C12A···H11W	2.75 (2)	H51D···O41D	2.7300
C12A···H11D	2.754 (17)	H51D···N41C^iii^	2.8300
C12A···H41C^iii^	2.93 (2)	H51D···H32D	2.5900
C12A···H61D^ii^	2.8100	H51D···H42C^iii^	2.3800
C12A···H6B	2.9400	H52D···C41C^ii^	2.9600
C12B···H41D^vi^	3.01 (2)	H52D···N41C^ii^	2.8300
C12B···H12D	2.75 (2)	H61C···C6A^xi^	3.0600
C12B···H12W^iv^	2.79 (3)	H61C···H6A^xi^	2.4000
			
H11W—O1W—H12W	107 (2)	H31D—C3D—H32D	108.00
C2C—N1C—C6C	112.75 (13)	C4D—C3D—H31D	109.00
C2C—N1C—H12C	109.7 (11)	C3D—C4D—H4D	109.00
C6C—N1C—H11C	104.3 (10)	C41D—C4D—H4D	109.00
H11C—N1C—H12C	114.0 (17)	C5D—C4D—H4D	109.00
C2C—N1C—H11C	111.4 (10)	C4D—C5D—H51D	109.00
C6C—N1C—H12C	104.5 (11)	C6D—C5D—H51D	109.00
H41C—N41C—H42C	117.5 (16)	C6D—C5D—H52D	109.00
C41C—N41C—H42C	121.2 (12)	H51D—C5D—H52D	108.00
C41C—N41C—H41C	121.2 (11)	C4D—C5D—H52D	109.00
C2D—N1D—C6D	112.54 (12)	N1D—C6D—H62D	109.00
C2D—N1D—H11D	110.3 (10)	C5D—C6D—H61D	109.00
C6D—N1D—H11D	109.8 (10)	N1D—C6D—H61D	109.00
C6D—N1D—H12D	107.7 (11)	H61D—C6D—H62D	108.00
H11D—N1D—H12D	109.6 (15)	C5D—C6D—H62D	109.00
C2D—N1D—H12D	106.8 (11)	C2A—C1A—C6A	117.89 (15)
C41D—N41D—H42D	119.1 (13)	C6A—C1A—C11A	120.65 (15)
H41D—N41D—H42D	122.7 (17)	C2A—C1A—C11A	121.41 (15)
C41D—N41D—H41D	118.0 (11)	C1A—C2A—C3A	120.69 (18)
N1C—C2C—C3C	110.40 (14)	C2A—C3A—C4A	120.9 (2)
C2C—C3C—C4C	111.32 (14)	C3A—C4A—C5A	119.43 (17)
C3C—C4C—C41C	111.47 (12)	C4A—C5A—C6A	120.04 (18)
C3C—C4C—C5C	109.65 (12)	C1A—C6A—C5A	121.08 (16)
C5C—C4C—C41C	110.33 (12)	C1A—C11A—C12A	109.48 (12)
C4C—C5C—C6C	111.06 (13)	O12A—C12A—C11A	117.85 (13)
N1C—C6C—C5C	111.00 (13)	O13A—C12A—C11A	117.78 (13)
O41C—C41C—C4C	121.01 (13)	O12A—C12A—O13A	124.35 (13)
O41C—C41C—N41C	122.48 (15)	C1A—C2A—H2A	120.00
N41C—C41C—C4C	116.51 (13)	C3A—C2A—H2A	120.00
N1C—C2C—H21C	110.00	C2A—C3A—H3A	120.00
N1C—C2C—H22C	110.00	C4A—C3A—H3A	120.00
C3C—C2C—H22C	110.00	C5A—C4A—H4A	120.00
H21C—C2C—H22C	108.00	C3A—C4A—H4A	120.00
C3C—C2C—H21C	110.00	C4A—C5A—H5A	120.00
C2C—C3C—H31C	109.00	C6A—C5A—H5A	120.00
C2C—C3C—H32C	109.00	C5A—C6A—H6A	120.00
C4C—C3C—H32C	109.00	C1A—C6A—H6A	119.00
C4C—C3C—H31C	109.00	C1A—C11A—H11A	110.00
H31C—C3C—H32C	108.00	C12A—C11A—H11A	110.00
C41C—C4C—H4C	108.00	C12A—C11A—H12A	110.00
C5C—C4C—H4C	108.00	H11A—C11A—H12A	108.00
C3C—C4C—H4C	108.00	C1A—C11A—H12A	110.00
C6C—C5C—H51C	109.00	C2B—C1B—C6B	117.49 (16)
C4C—C5C—H51C	109.00	C2B—C1B—C11B	120.95 (15)
H51C—C5C—H52C	108.00	C6B—C1B—C11B	121.55 (15)
C6C—C5C—H52C	109.00	C1B—C2B—C3B	121.63 (16)
C4C—C5C—H52C	109.00	C2B—C3B—C4B	120.25 (17)
N1C—C6C—H61C	109.00	C3B—C4B—C5B	118.79 (17)
C5C—C6C—H62C	109.00	C4B—C5B—C6B	121.05 (16)
N1C—C6C—H62C	109.00	C1B—C6B—C5B	120.79 (15)
C5C—C6C—H61C	109.00	C1B—C11B—C12B	113.90 (14)
H61C—C6C—H62C	108.00	O12B—C12B—C11B	117.73 (14)
N1D—C2D—C3D	111.01 (12)	O13B—C12B—C11B	118.34 (14)
C2D—C3D—C4D	111.40 (12)	O12B—C12B—O13B	123.92 (14)
C5D—C4D—C41D	111.99 (12)	C3B—C2B—H2B	119.00
C3D—C4D—C5D	109.56 (12)	C1B—C2B—H2B	119.00
C3D—C4D—C41D	109.57 (12)	C4B—C3B—H3B	120.00
C4D—C5D—C6D	111.24 (12)	C2B—C3B—H3B	120.00
N1D—C6D—C5D	111.19 (12)	C3B—C4B—H4B	121.00
N41D—C41D—C4D	116.31 (13)	C5B—C4B—H4B	121.00
O41D—C41D—N41D	122.36 (15)	C4B—C5B—H5B	119.00
O41D—C41D—C4D	121.32 (13)	C6B—C5B—H5B	120.00
N1D—C2D—H22D	109.00	C5B—C6B—H6B	120.00
C3D—C2D—H21D	109.00	C1B—C6B—H6B	120.00
C3D—C2D—H22D	109.00	C1B—C11B—H11B	109.00
H21D—C2D—H22D	108.00	C1B—C11B—H12B	109.00
N1D—C2D—H21D	109.00	C12B—C11B—H12B	109.00
C2D—C3D—H31D	109.00	H11B—C11B—H12B	108.00
C2D—C3D—H32D	109.00	C12B—C11B—H11B	109.00
C4D—C3D—H32D	109.00		
			
C6C—N1C—C2C—C3C	56.10 (17)	C6A—C1A—C2A—C3A	0.8 (2)
C2C—N1C—C6C—C5C	−56.36 (17)	C11A—C1A—C2A—C3A	−176.53 (15)
C6D—N1D—C2D—C3D	55.70 (16)	C2A—C1A—C6A—C5A	−1.5 (2)
C2D—N1D—C6D—C5D	−55.55 (17)	C11A—C1A—C6A—C5A	175.77 (14)
N1C—C2C—C3C—C4C	−55.83 (18)	C2A—C1A—C11A—C12A	95.07 (17)
C2C—C3C—C4C—C41C	178.39 (13)	C6A—C1A—C11A—C12A	−82.13 (16)
C2C—C3C—C4C—C5C	55.91 (17)	C1A—C2A—C3A—C4A	0.4 (3)
C3C—C4C—C41C—O41C	−58.46 (19)	C2A—C3A—C4A—C5A	−0.7 (3)
C3C—C4C—C41C—N41C	121.61 (15)	C3A—C4A—C5A—C6A	0.0 (3)
C5C—C4C—C41C—O41C	63.62 (19)	C4A—C5A—C6A—C1A	1.2 (2)
C5C—C4C—C41C—N41C	−116.31 (15)	C1A—C11A—C12A—O12A	−87.75 (17)
C3C—C4C—C5C—C6C	−55.69 (17)	C1A—C11A—C12A—O13A	90.43 (16)
C41C—C4C—C5C—C6C	−178.84 (12)	C6B—C1B—C2B—C3B	0.8 (3)
C4C—C5C—C6C—N1C	55.90 (17)	C11B—C1B—C2B—C3B	179.74 (17)
N1D—C2D—C3D—C4D	−55.88 (16)	C2B—C1B—C6B—C5B	−1.1 (3)
C2D—C3D—C4D—C5D	55.72 (16)	C11B—C1B—C6B—C5B	179.96 (17)
C2D—C3D—C4D—C41D	178.95 (12)	C2B—C1B—C11B—C12B	−54.1 (2)
C3D—C4D—C5D—C6D	−55.38 (16)	C6B—C1B—C11B—C12B	124.84 (17)
C41D—C4D—C5D—C6D	−177.17 (12)	C1B—C2B—C3B—C4B	0.1 (3)
C3D—C4D—C41D—O41D	−66.13 (18)	C2B—C3B—C4B—C5B	−0.6 (3)
C3D—C4D—C41D—N41D	112.70 (15)	C3B—C4B—C5B—C6B	0.3 (3)
C5D—C4D—C41D—O41D	55.66 (19)	C4B—C5B—C6B—C1B	0.6 (3)
C5D—C4D—C41D—N41D	−125.52 (15)	C1B—C11B—C12B—O12B	−53.1 (2)
C4D—C5D—C6D—N1D	55.47 (16)	C1B—C11B—C12B—O13B	127.76 (16)

Symmetry codes: (i) *x*−1, −*y*+1/2, *z*+1/2; (ii) *x*, −*y*+1/2, *z*+1/2; (iii) *x*, *y*, *z*+1; (iv) *x*, −*y*+1/2, *z*−1/2; (v) *x*−1, *y*, *z*+1; (vi) *x*−1, *y*, *z*; (vii) *x*, *y*, *z*−1; (viii) −*x*+1, −*y*, −*z*+2; (ix) *x*+1, *y*, *z*; (x) *x*+1, −*y*+1/2, *z*−1/2; (xi) *x*+1, *y*, *z*−1; (xii) −*x*, −*y*, −*z*+1; (xiii) −*x*+1, −*y*, −*z*+1.

### Hydrogen-bond geometry (Å, °)

**Table:**

*D*—H···*A*	*D*—H	H···*A*	*D*···*A*	*D*—H···*A*
N1C—H11C···O13B^ix^	0.941 (18)	1.826 (18)	2.7638 (17)	174.8 (17)
N1C—H12C···O13A^xi^	0.93 (2)	1.85 (2)	2.7322 (18)	157.9 (18)
N1D—H11D···O12A	0.924 (17)	1.876 (17)	2.7871 (17)	168.4 (16)
N1D—H12D···O12B	0.96 (2)	1.82 (2)	2.7095 (18)	153.0 (17)
N41C—H41C···O12A^vii^	0.86 (2)	2.03 (2)	2.8789 (19)	166.3 (16)
N41C—H42C···O41D^vii^	0.938 (18)	1.918 (18)	2.8480 (18)	170.8 (14)
N41D—H41D···O13B^ix^	0.87 (2)	2.08 (2)	2.9177 (19)	160.6 (16)
N41D—H42D···O41C	0.913 (19)	1.917 (19)	2.8294 (18)	176.4 (18)
O1W—H11W···O13A	0.85 (2)	1.95 (2)	2.7881 (19)	171 (2)
O1W—H12W···O12B^ii^	0.84 (3)	1.99 (3)	2.8335 (19)	179 (3)
C6B—H6B···O13A	0.93	2.48	3.349 (2)	156

Symmetry codes: (ix) *x*+1, *y*, *z*; (xi) *x*+1, *y*, *z*−1; (vii) *x*, *y*, *z*−1; (ii) *x*, −*y*+1/2, *z*+1/2.
